# Editorial: Recent advances and new biomarkers in ulcerative colitis, volume II

**DOI:** 10.3389/fmed.2025.1657870

**Published:** 2025-07-17

**Authors:** Tsvetelina Velikova, Xiang Xue

**Affiliations:** 1 Medical Faculty, Sofia University St. Kliment Ohridski, Sofia, Bulgaria; 2 Department of Biochemistry and Molecular Biology, University of New Mexico, Albuquerque, NM, United States

**Keywords:** ulcerative colitis, Th17/Treg cells, mucosal healing, atorvastatin, biomarkers, anoikis gene

The topic of identifying new biomarkers for ulcerative colitis (UC) that could improve not only our understanding of inflammatory bowel disease pathogenesis, but also revolutionize the diagnosis, prognosis prediction, and treatment of this chronic inflammatory condition, remains a hot topic in Gastroenterology. After the initial issues of the special collection, we gathered additional impactful papers on the topic, which investigate practical aspects such as measuring the C-reactive protein (CRP)-to-albumin ratio and the neutrophil-to-albumin ratio for predicting therapy response, or determining an anakinra-related gene signature, along with some new advances in Th17/Treg cell balance and mucosal healing.

Since UC is a chronic, relapsing inflammatory bowel disease marked by progressive mucosal damage, the community still questions what's new. Zhao et al., in their review, focused on the mucosal immune dysregulation, genetic mutations, and environmental triggers that compromise the intestinal barrier, enabling pathogenic bacteria and antigens to enter the lamina propria and intensify inflammation. They demonstrated new insights into the key role of the imbalance between proinflammatory Th17 cells and regulatory T cells (Treg), characterized by elevated Th17 activity and reduced Treg-mediated suppression. Furthermore, they emphasized the importance of maintaining the Th17/Treg balance in managing inflammation and immune response in UC, summarizing evidence from clinical and experimental studies on the ability of plant-based substances to regulate Th17/Treg dynamics in UC. These findings support the development of novel, plant-based therapeutic agents for future clinical applications in the treatment of UC.

Delving more into the UC pathogenesis, Liu et al. demonstrated that anoikis-related genes (ARGs) play a significant role. By integrating Gene Expression Omnibus datasets (GSE75214, GSE92415, and GSE16879) and identifying 58 ARGs through the gene set enrichment analysis, the authors applied machine learning algorithms—LASSO Cox regression, random forest, and support vector machine—to identify five key anoikis differentially expressed genes (DEGs): PDK4, CEACAM6, CFB, CX3CL1, and HLA-DMA. Receiver operating characteristic analysis confirmed their high diagnostic accuracy. CEACAM6, CFB, CX3CL1, and HLA-DMA were positively correlated with immune cell infiltration, while PDK4 showed negative associations. Unsupervised clustering divided UC patients into two subtypes with distinct gene expression and immune pathway profiles. Further regulatory network analysis identified TP53, RARB, RXRB, and CTCF as potential upstream regulators of the pathway. This comprehensive analysis revealed the clinical relevance of anoikis-DEGs as immune-associated biomarkers in UC, offering novel insights into disease mechanisms and supporting their potential as targets for personalized diagnostic and therapeutic strategies.

In line with this, another original paper by Alarfaj et al. showed that high-dose atorvastatin significantly attenuates inflammation and improves symptoms in patients with mild to moderate UC when used as an adjuvant to mesalamine. In this randomized, controlled pilot study, patients received either mesalamine alone or in combination with atorvastatin (80 mg daily) for six months. Compared to the placebo group, the atorvastatin group exhibited significant reductions in proinflammatory markers, including IL-6, S1P, TNF-α, nitric oxide, CRP, erythrocyte sedimentation rate (ESR), and fecal calprotectin. Clinical outcomes also improved, with greater decreases in partial Mayo scores and enhanced quality of life scores (SF-36). Response and remission rates were higher in the atorvastatin group (91.3% and 60.8%, respectively) compared to placebo (83.3% and 45.8%). These findings suggest that atorvastatin may exert beneficial effects by modulating the S1P/TNF-α/IL-6 pathway, supporting its potential role as an adjunctive therapy in the management of UC. These findings inform future research in the field.

Logically, we must ask ourselves—do we have sufficient surrogate markers to evaluate mucosal healing, or are we still struggling? More in-depth, Jin et al. reviewed the concept of deep mucosal healing (MH) in UC, extending the vision beyond endoscopic remission. Moreover, MH has become a critical therapeutic target in the pursuit of long-term disease control. While endoscopic remission is widely used in clinical practice, it is subject to interobserver variability. Increasing evidence supports that histological remission may offer better prognostic value, including reduced relapse rates, although its routine use as a therapeutic endpoint remains debated. The authors highlighted the evolving definitions of MH across various endoscopic and histological scoring systems, emphasizing the lack of a universal, standardized definition for deep MH. Technological advances have significantly enriched our understanding of mucosal healing; however, a consensus is still lacking. However, the authors argue that integrating both endoscopic and histological markers may improve long-term outcomes in UC. Large-scale prospective studies are warranted to validate the significance of deep MH further and clarify its role in guiding treatment decisions.

Zhang et al. demonstrated that the CRP-to-albumin ratio (CAR) and neutrophil-to-albumin ratio (NAR) are promising biomarkers for predicting treatment response and long-term prognosis in UC patients receiving infliximab therapy. In a cohort of 157 UC patients and 199 controls, CAR and NAR were measured before treatment, after induction, and at 8-week intervals over 54 weeks. Statistical analyses revealed that both ratios correlated significantly with disease activity. Importantly, elevated CAR and NAR values were associated with lower clinical response rates after infliximab induction and reduced likelihood of achieving clinical remission at week 54. The study showed that both indices can serve as early indicators of treatment efficacy and provide valuable prognostic insight throughout the infliximab treatment course. These findings support the utility of CAR and NAR as accessible, non-invasive markers for guiding therapeutic decisions and monitoring disease progression in patients with UC undergoing biologic therapy.

In conclusion, this collection highlights emerging biomarkers and therapeutic strategies for ulcerative colitis, ranging from immunological pathways, such as the Th17/Treg balance and anoikis-related genes, to practical tools, including CAR and NAR. Together, these studies emphasize the need for reliable surrogate markers to guide personalized treatment and deepen our understanding of mucosal healing in UC.

